# P-2186. Implementing Updated Hepatitis B Virus Screening Guidelines in an Underserved Resident Clinic

**DOI:** 10.1093/ofid/ofae631.2340

**Published:** 2025-01-29

**Authors:** Cole J White, John C Weis, Mariah P Barlow

**Affiliations:** OhioHealth Riverside Methodist Hospital, Columbus, Ohio; OhioHealth Riverside Methodist Hospital, Columbus, Ohio; OhioHealth Riverside Methodist Hospital, Columbus, Ohio

## Abstract

**Background:**

An estimated 580,000 to 2.4 million Americans are living with hepatitis B virus (HBV) infection putting them at increased risk of cirrhosis, hepatocellular carcinoma, and death. Given these adverse outcomes, the Centers for Disease Control and Prevention recommended universal hepatitis B screening with a triple panel for all adults aged 18 and older starting in 2023. The purpose of our quality improvement (QI) project was to increase HBV screening rates in our internal medicine residency program outpatient clinic in accordance with updated CDC recommendations.

Table 1

**Figure:**
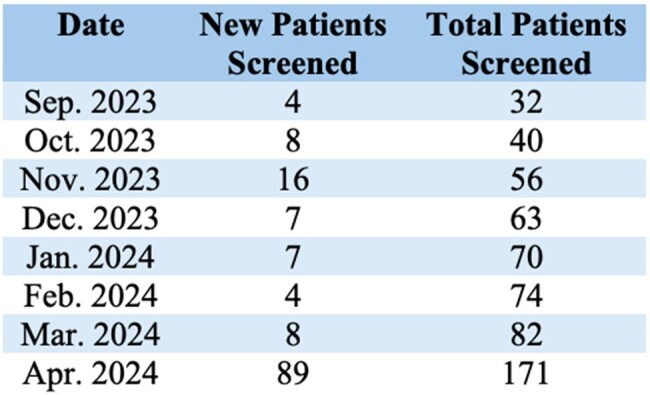

The number of new patients screened for HBV and the total number of patients screened for HBV, organized by month.

**Methods:**

This QI project took place at a single outpatient internal medicine residency clinic from August 2023 to April 2024. HBV screening rates were obtained at baseline and monthly thereafter. All patients 18 years and older were included unless prior billing and coding data demonstrated a diagnosis of hepatitis B. 2798 patients were identified in the population at the beginning of the study. Patients were deemed as successfully screened if they had a documented HBV triple screen [hepatitis B surface antigen (HBsAg), antibody to hepatitis B surface antigen (anti-HBs), and antibody to hepatitis B core antigen (anti-HBc)]. Two Plan-Do-Study-Act (PDSA) cycles were implemented; the first consisted of resident education and the second was the creation of an electronic medical record (EMR) reminder.

Figure 1

**Figure:**
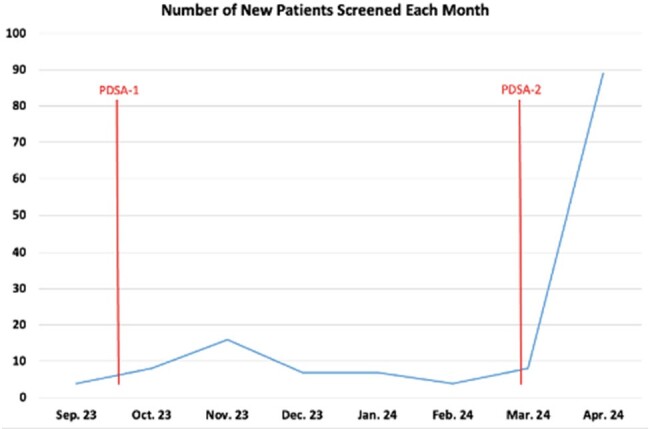

The number of patients screened for HBV by month.

**Results:**

Only 32 patients were screened for HBV at baseline. In the six months following PDSA-1, 50 patients were screened. This brought the total number of patients screened to 82, representing a 156% increase in HBV screening after PDSA-1. In the month following the start of PDSA-2, 89 additional patients were screened for HBV, increasing the overall number screened to 171.

Figure 2

**Figure:**
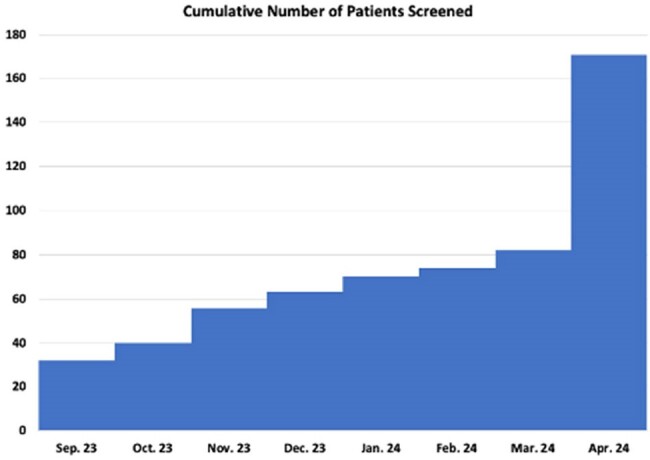

The cumulative number of patients that have been screened for HBV, measured monthly.

**Conclusion:**

PDSA-1 (resident education) showed an initial increase in HBV screening followed by a slow decline in screening over time. This suggests that education may transiently increase screening rates; however, screening will decline over time as the recency effect abates. PDSA-2 (EMR reminder) led to a more rapid increase in screening rates which suggests this may be a more impactful way to quickly identify patients in need of HBV screening. Ongoing data collection is needed to determine if EMR-generated reminders are capable of sustaining screening rates over time.

**Disclosures:**

All Authors: No reported disclosures

